# Unveiling potential diagnostic biomarkers for rheumatoid arthritis through integrated gene expression analysis

**DOI:** 10.3389/fimmu.2026.1645257

**Published:** 2026-02-24

**Authors:** Zhi-wei Feng, Ming-kun Yang, Xin-dong Jia, Fa Yuan, Ming-gang Guo, Feng Chen, Wei Li, Chen-fei Yang

**Affiliations:** 1 Department of Orthopedics, Bazhong Central Hospital, Bazhong, China; 2 Department of Orthopaedics, Nanchong Central Hospital, The Second Clinical Institute of North Sichuan Medical College, Nanchong, China; 3 School of Nursing, North Sichuan Medical College, Nanchong, China

**Keywords:** diagnostic biomarker, immune infiltration, machine learning, rheumatoid arthritis, WGCNA

## Abstract

**Objective:**

Rheumatoid arthritis (RA) is a chronic autoimmune disorder that significantly impacts quality of life. Despite extensive research, its pathogenesis remains unclear. This study aims to identify potential diagnostic biomarkers and therapeutic targets for RA.

**Methods:**

This study integrated patient data from three Gene Expression Omnibus (GEO) databases to analyze gene expression in RA. Using Weighted Gene Correlation Network Analysis (WGCNA), we identified key genes, which were then compared with differentially expressed genes (DEGs) to uncover RA-related genes. Functional enrichment analysis provided insights into the biological roles of these genes. To refine our findings, we applied three algorithms—RandomForest, SVM-REF, LASSO, and Convolutional Neural Networks (CNN)—to pinpoint a subset of core genes. We evaluated their diagnostic potential through receiver operating characteristic (ROC) curves and selected the top five genes with the highest area under the curve (AUC) values for constructing a predictive nomogram model. An interaction analysis was performed to investigate the relationship between these genes and immune cell infiltration. Finally, the expression of these core genes was validated in the synovial tissues of RA patients. Drug-protein interaction relationships were predicted using the DSigDB database.

**Results:**

Differential expression analysis identified 543 DEGs. We subsequently applied WGCNA to compare these DEGs with significant module genes, resulting in the identification of 273 key genes. Functional enrichment analysis indicated that these genes were primarily involved in inflammatory response pathways. Further analysis using four machine learning algorithms identified 11 core genes. Of these, the five genes with the highest AUC values were selected to construct a robust nomogram model. Immune infiltration analysis revealed significant differences in immune cell levels and pathways between RA patients and healthy controls, which were correlated with the expression of these five genes. Validation through quantitative real-time PCR (qRT-PCR), Western blot, and immunofluorescence (IF) confirmed that GABARAPL1, FKBP5, and PCDH9 expression was lower in RA synovial tissues compared to healthy controls, while SLAMF8 expression was elevated. Additionally, potential therapeutic drugs targeting these key genes, including (+)-chelidonine, daunorubicin, and bisacodyl, were predicted.

**Conclusion:**

GABARAPL1, FKBP5, PCDH9, and SLAMF8 are identified as potential biomarkers for RA, offering insights into future therapeutic strategies.

## Introduction

Rheumatoid arthritis (RA) is a chronic autoimmune disease marked by systemic inflammation and persistent synovitis (1–3). The pathology of RA involves immune cell infiltration, hyperplasia of the synovial lining, pannus formation, and destruction of articular cartilage and bone (4). Currently, RA affects approximately 0.5–1% of adults worldwide, imposing significant economic burdens on both society and patients (5). Females are particularly more susceptible to RA, with a risk two to three times higher than that of males (6). Symptoms of RA include morning stiffness, which, if untreated, can progress to focal necrosis, granulation adhesion, and fibrous tissue formation on joint surfaces. This progression may lead to severe joint ankylosis, destruction, deformities, and disability (7). Researches show genetic, environmental, and immune factors are recognized to play significant roles (1, 8), despite extensive research efforts, the complete pathogenesis of RA remains elusive. Furthermore, current diagnostic approaches for RA are inadequate, underscoring the critical need for the development of reliable and efficient biomarkers to improve patient outcomes (9).

The etiology and pathogenesis of RA involve a multifaceted interplay of infectious agents, genetic susceptibilities, and environmental factors that trigger immune responses, resulting in synovitis. Studies indicate that the progression of RA is greatly affected by the atypical morphology and gene expression observed in RA macrophages (RASM) and synovial fibroblasts (RASF) (10). B cells exacerbate RA by producing proteins such as rheumatoid factor (RF), anti-citrullinated protein antibodies (ACPA), and pro-inflammatory cytokines. These proteins form immune complexes with self-antigens, thereby sustaining the disease process (11). Upon stimulation by antigen-presenting cells, CD4+ T cells evolve into five distinct lineages of T helper (Th) cells—Th17, Th2, Th1, T follicular helper (Tfh), and T regulatory (Treg) cells (12). In RA, these T cells predominantly activate macrophages and fibroblasts, promoting their transformation into cells that damage tissues (13). Despite this understanding, the precise mechanisms underlying gene and protein expression in RA-associated synovium remain poorly understood.

The primary therapeutic options for RA include anti-inflammatory drugs, analgesics, and disease-modifying antirheumatic drugs (DMARDs). Anti-inflammatory drugs and analgesics only relieve RA symptoms without stopping disease progression. Because immune responses are crucial in the pathogenesis of RA, DMARDs are the preferred treatment method. Although DMARDs effectively reduce RA symptoms, many patients still experience treatment failures, including nonresponse or limited effectiveness (14). Over the last decade, biological agents have increasingly been tested in clinical trials. These agents target immune cells for immunomodulation and are often used alongside DMARDs to manage RA (15). Whether used alone or combined with new biologics, the optimal treatment regimen for RA is still being researched. Thus, exploring genes linked to the onset and progression of RA is essential for enhancing early diagnosis and developing better therapeutic strategies.

Substantial attention is currently focused on identifying disease-specific biomarkers using bioinformatics and genome sequencing technologies (16). Our research aimed to pinpoint key biomarkers related to DEGs and immune infiltration in RA, with the ultimate goal of developing novel diagnostic and therapeutic strategies for this condition.

## Materials and methods

### Data processing

Microarray datasets for RA were retrieved from the GEO database using the following criteria: “Rheumatoid Arthritis,” “Expression profiling by array,” “Homo sapiens,” and “sample count >20.” Three gene expression profiles (GSE12021, GSE55457, and GSE55235) based on the GPL96 platform were selected for further analysis. GSE12021 includes 12 synovial tissue samples from RA patients and 9 from healthy knee joints. GSE55457 consists of 23 samples, with 10 from healthy synovial tissue and 13 from RA patients. GSE55235 contains 20 samples, 10 from healthy knee joints and 10 from RA patients. Furthermore, GSE77298 was used as an independent dataset because the validation group (including RA/normal synovial samples) consisted of 16/7 samples, respectively. Using the “SVA” software, we eliminated batch effects and normalized the data (17). Specifically, we re-analyzed the three datasets by combining the expression matrices from all three arrays before applying the normalization process. were then identified using the “Limma” package (18). The criteria for DEGs identification included an adjusted *P*-value < 0.05 and |logFC| > 1. Expression heat maps were generated using the “pheatmap” package.

### Construction of WGCNA

The WGCNA method (19) is employed to explore the relationships between gene sets and clinical traits by constructing gene co-expression networks. Using the “WGCNA” R package, gene networks were constructed and modularized in several stages. WGCNA was applied to all genes that passed quality control (QC), not just DEGs. Initially, samples were clustered to identify significant outliers. Automated procedures were then used to construct the co-expression networks. Modules were identified through hierarchical clustering combined with dynamic tree cutting. To correlate modules with clinical traits, module membership (MM) and gene significance (GS) were calculated. Hub modules were defined by the highest Pearson correlation of MM and a *P*-value ≤ 0.05. High connectivity within modules and their clinical relevance were determined by MM values > 0.8 and GS values > 0.2, respectively. The network was constructed as an unsigned network to capture both positive and negative correlations between genes. The primary goal of this method is to identify key modules and genes that are strongly associated with clinical traits, which could serve as potential biomarkers for further investigation.

### Functional analysis

Functional enrichment analysis was performed to clarify the potential functions of prospective targets. Gene Set Enrichment Analysis (GSEA) (20) was performed using the immunologic signature gene set (C7 gene sets) from the Molecular Signatures Database (MSigDB, https://www.gsea-msigdb.org/) using the “clusterProfiler” package. Gene ontology (GO) is commonly used to assign functions to genes, particularly in terms of biological pathways (BP), molecular functions (MF), and cellular components (CC). KEGG (21) enrichment analysis offers insights into gene functions and associated high-level genomic information. The R packages “GOplot” and “clusterProfiler” were used to explore the GO functions of potential mRNAs and improve KEGG pathways, leading to a better comprehension of the carcinogenic implications of target genes (22).

### Identification of hub genes

Feature genes, crucial for diagnosing RA, were isolated from previously identified genes. SVM-RFE, a machine learning technique, was employed to train a subset of features (genes) across different categories (such as patient vs. healthy groups), identifying the most predictive genes for RA diagnosis (23). The “glmnet” package in R was used to perform LASSO regression, which aims to select linear models by shrinking coefficients of less important variables to zero, thereby retaining the most relevant features for predictive modeling. LASSO classification, utilizing binomial distribution variables, was performed with a lambda value set at the 1-SE criterion, achieving optimal performance with only 10 cross-validation variables. RandomForest ranked the genes, with values above 0.25 considered significant (24). To further strengthen the analysis, advanced machine learning models, including CNN, were also applied. The CNN model was implemented using Python to capture complex patterns in gene expression data, offering a deep learning approach. The performance of CNN was compared to traditional machine learning models, assessing its ability to distinguish RA patients from healthy controls. The intersection of results from LASSO regression, SVM-RFE, RandomForest, and CNN analysis revealed the most significant feature genes in this study.

### Curve analysis of ROC

The “pROC” package was used to compute the area under the curve (AUC), generate Receiver Operating Characteristic (ROC) curves, and assess the diagnostic usefulness of signature genes in separating RA cases from normal samples using the GSE77298 dataset (25).

### Development and validation of diagnostic nomogram

A nomogram model predicting RA recurrence was constructed using the “rms” package. In this model, “score” denotes the score for each variable, while “total score” represents the cumulative sum of all individual scores. Using calibration curves, the prediction accuracy of the model was evaluated. Clinical impact curves and decision curve analysis were used to assess the model’s clinical usefulness.

### Construction of PPI network

GeneMANIA (26) (http://www.genemania.org) enables the creation of Protein–Protein Interaction (PPI) networks, facilitating the prediction of gene functions and identification of genes with similar effects. Many bioinformatics techniques, including physical interactions, co-expression, co-localization, gene enrichment analysis, genetic relationships, and site predictions, are used by this network integration program. GeneMANIA was used in this research to examine the PPI networks including signature genes.

### Immune infiltration analysis using ssGSEA

Single-sample gene set enrichment analysis (ssGSEA) (20) was used to quantify the infiltration scores of 16 immune types and the activities of 13 immune-related pathways. The infiltration score represents the relative abundance of each immune cell type or pathway in a given sample. The Spearman rank correlation coefficient was computed using the “corrplot” software to investigate the association between immunological state and certain genes.

### Clinical specimens

Synovial tissue samples were collected from nine patients diagnosed with RA at Nanchong Central Hospital, all of whom met the clinical and imaging diagnostic criteria for RA. Additionally, nine young patients with no history of RA and undergoing surgical treatment for meniscal injuries, provided synovial tissue samples as trauma controls (TC). The inclusion criteria for the RA group required patients to meet both clinical and imaging diagnostic criteria for RA, while those with other autoimmune diseases or infections were excluded. For the TC group, inclusion was restricted to patients without a history of RA, and individuals with inflammatory joint diseases or autoimmune disorders were excluded. Sample IDs and demographic details for both groups are outlined in **Supplementary Table 1**.

### RNA extraction and qRT-PCR

To extract total RNA, synovial tissue was treated with Trizol reagent (Invitrogen, Carlsbad, CA, USA). After the RNA was separated, it was reverse transcribed into complementary DNA (cDNA) using gDNA Eraser and the PrimeScript RT Reagent Kit from Takara Bio, Inc. in Kyoto, Japan. SYBR Green qPCR Mix (Takara Bio, Inc., Japan) was used for qRT-PCR tests, which were performed using CFX96 quantitative PCR equipment (Bole, Inc., California, USA). Target gene expression levels were measured using the 2^-ΔΔCq^ method, normalized to GAPDH (27). **Table 1** contains a collection of qRT-PCR primer sequences.

**Table 1:** The sequence of primers.

Gene	Forward and reverse primer
FKBP5	F:5’ GCGAAGGAGAAGACCACGACAT 3’
	R:5’ TAGGCTTCCCTGCCTCTCCAAA 3’
GABARAPL1	F:5’ CCCTCCCTTGTTATCATCCA 3’
	R:5’ ACTCCCACCCCACAAAATCC 3’
MREG	F:5’ CCCTTGGCATTTTATCTGGA 3’
	R:5’ AAGCTGCATTCACAGCATTG 3’
PCDH9	F:5’ TCCCAACTCTGATGGGCCTTTGGG 3’
	R:5’ GGCTCTGGTCAGGGTGTGCC 3’
SLAMF8	F:5’ CTGATGGTGGATACAAGGG 3’
	R:5’ GGAAATGGACGTAACGGA 3’
GAPDH	F:5’ TGTGTCCGTCGTGGATCTGA 3’ R:5’ TTGCTGTTGAAGTCGCAGGAG 3’

### Protein extraction and Western blot

Proteins were extracted from synovial tissue using RIPA buffer (Beyotime, China) and quantified with BCA Protein Assay Kits (Beyotime). After that, the proteins were heated for 10 minutes and mixed in a 1:3 ratio with loading buffer to denature them. The proteins were separated using SDS-PAGE and then placed onto PVDF membranes (Millipore, USA). The PVDF membranes were subjected to an hour of blocking with 5% BSA at 4 °C. Subsequently, they were incubated with the primary antibodies against PCDH9 (Abcam, ab233710), GABARAPL1 (Abcam, ab109364), FKBP5 (Proteintech, 14155-1-AP), SLAMF8 (Novus Biologicals, AF1907), and GAPDH (Proteintech, 10494-1-AP) for an overnight period at 4°C. Membranes were incubated with the primary antibody for one hour at room temperature, and then treated with horseradish peroxidase-linked secondary antibody (Proteintech, 1:5000). An ECL kit (Biosharp, China) was used to observe the signal bands, and ImageJ software (Bethesda, MD, USA) was used to analyze the signal bands for gray values.

### Hematoxylin-eosin staining

Synovial tissue samples were taken and preserved for 48 hours in 10% neutral buffered formalin. The paraffin-embedded fixed tissues were sectioned after being fixed. Hematoxylin and eosin were used to stain the sections after they had been deparaffinized in xylene and progressively dried in ethanol. Sections were counterstained with eosin after differentiation. After being dried in graded ethanol, the dyed slices were mounted using neutral resin. The specimens were then studied using an Olympus fluorescent microscope (Japan).

### Immunofluorescence staining

This investigation validated diagnostic biomarkers using immunofluorescence (IF) labeling. To retrieve antigens, deparaffinized synovial tissue slices were soaked in a 10 mM citrate buffer solution (Solarbio, China). The sections were incubated with the primary antibody solution overnight at 4°C after being blocked for an hour with 5% BSA (Solarbio). The slices were incubated with a fluorescence-labeled secondary antibody solution (Proteintech) for an extra hour the following day. After incubating the sections, they were cleaned three times for ten minutes each using 1× TBST solution. After the nuclei were counterstained with the blue fluorescent dye DAPI, the coverslip was sealed with an anti-fluorescence quencher. After that, the samples were examined and captured on camera using an Olympus fluorescent microscope (Japan).

### Clinical correlation analysis

To assess the clinical relevance of the identified biomarkers, we examined the associations between gene expression levels measured by quantitative PCR (qPCR) and serum inflammatory indices within the rheumatoid arthritis (RA) cohort. Synovial tissue–derived qPCR expression values for the candidate genes (e.g., GABARAPL1, FKBP5, PCDH9, and SLAMF8) were matched to the corresponding clinical data from the same patients. ESR (mm/h) and CRP (mg/L) values were collected from routine laboratory testing at the time of sample acquisition. Only RA patients with available ESR and CRP measurements were included in the correlation analysis; cases with missing values were excluded listwise.

### Evaluation of applicant drugs

Protein-drug interaction networks play a critical role in drug development (28). In this study, we used the Enrichr tool (29) to analyze transcriptomic signatures from the DSigDB database (30), identifying potential drug candidates targeting core genes. The ten most promising drugs were selected based on their *P*-values. These drugs were recommended for targeting hub genes due to their potential therapeutic benefits for RA.

### Statistical analysis

Statistical analyses were performed using Prism 8.0 (GraphPad Software, San Diego, CA, USA). Normality was assessed using the Shapiro–Wilk test. For comparisons between two groups, an unpaired two-tailed Student’s t test was applied when data were normally distributed; otherwise, the non-parametric Mann–Whitney U test was used. For comparisons among multiple groups, one-way ANOVA with appropriate *post hoc* testing was performed for normally distributed data with homogeneity of variance; otherwise, the Kruskal–Wallis test followed by Dunn’s multiple-comparison test was applied. Data are presented as mean ± standard deviation (SD) for normally distributed variables and as median (interquartile range, IQR) for non-normally distributed variables. A two-sided *p* value < 0.05 was considered statistically significant. To ensure robustness and repeatability, all *in vitro* experiments were conducted in triplicate.

## Results

### DEGs screening and data preprocessing

**Figure 1:**
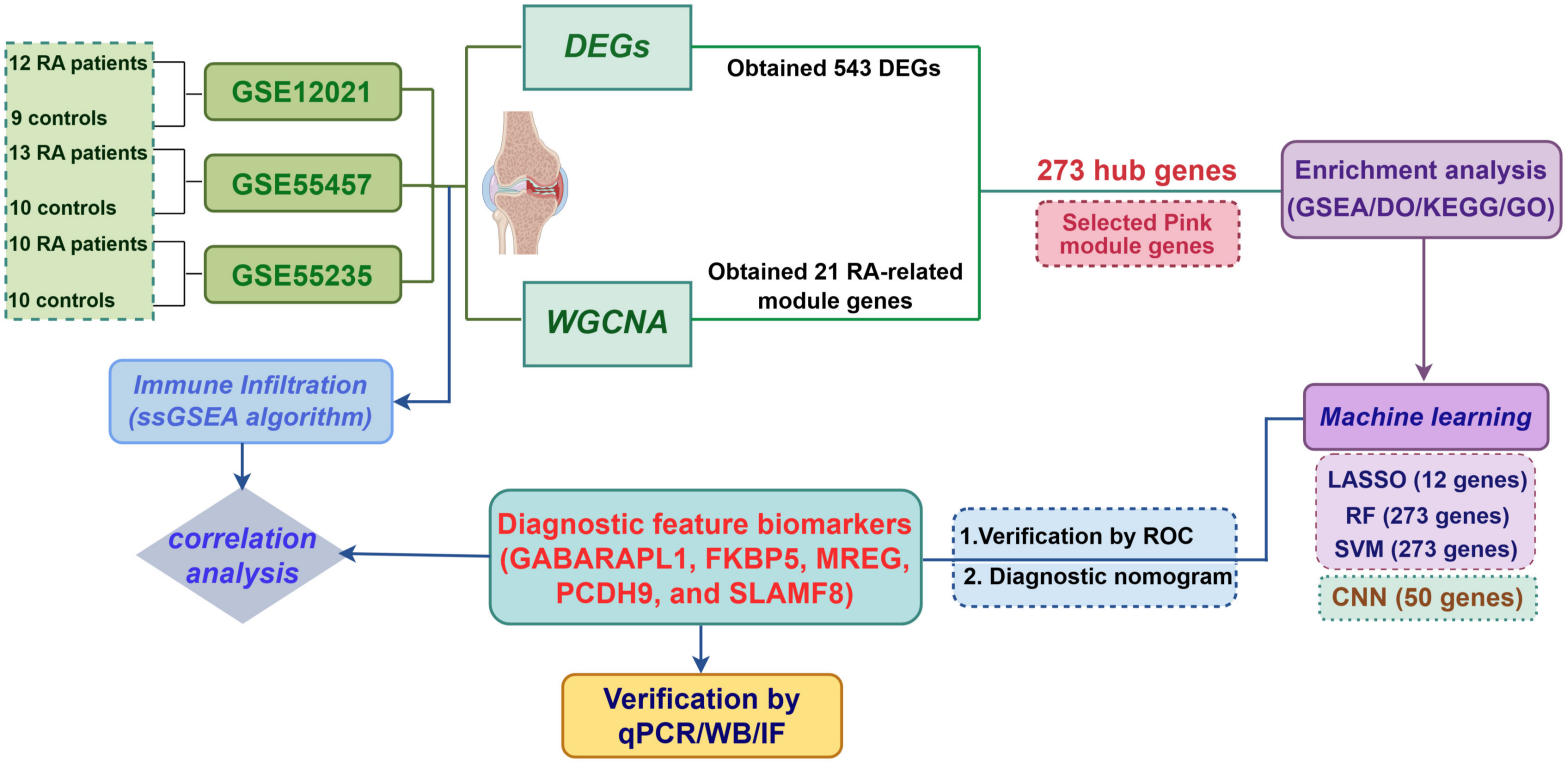
Flowchart depicting the comprehensive strategy for bioinformatics analyses.

**Figure 2:**
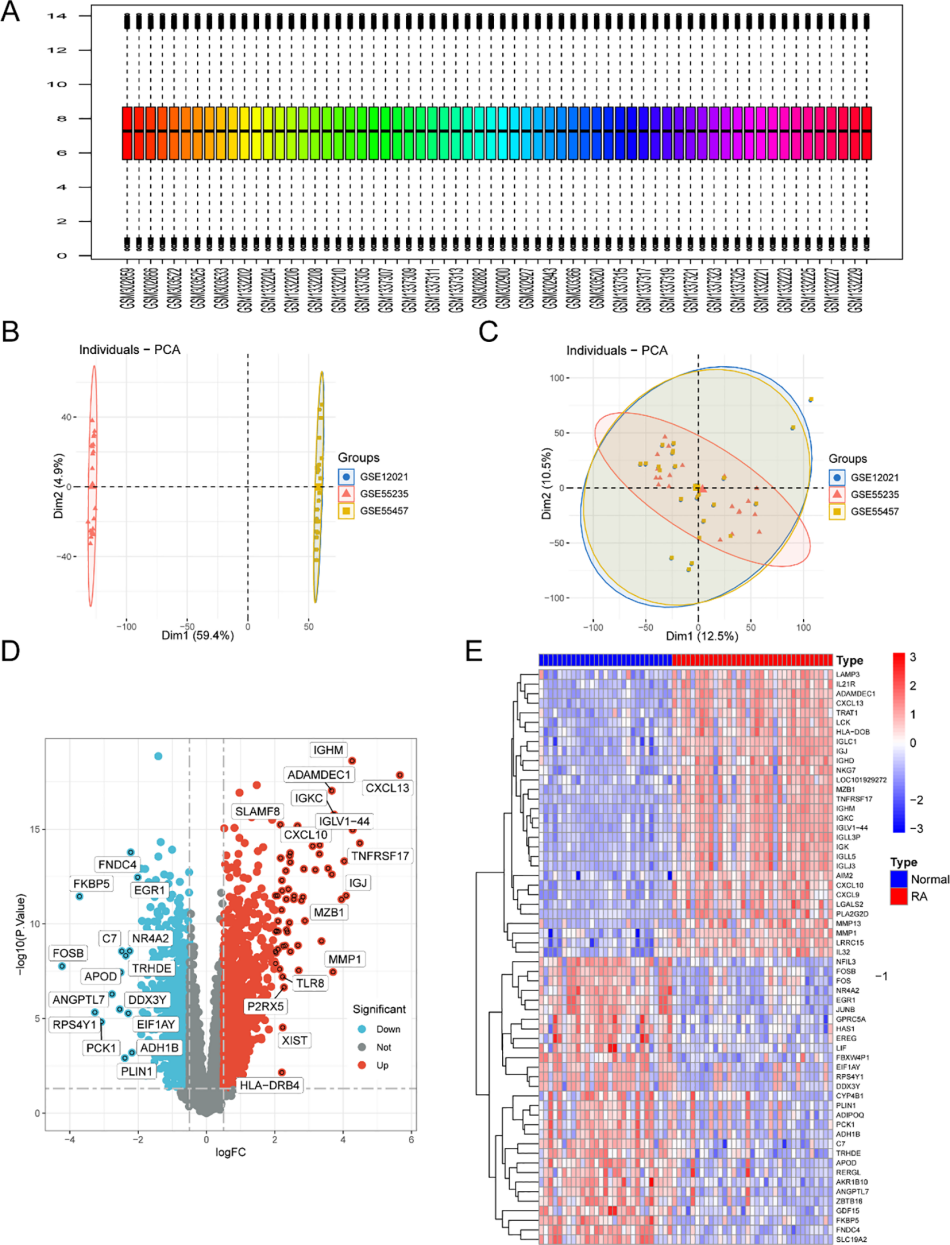
Data preprocessing for the DEGs. **(A)** Box plots depicting normalized raw data across samples. **(B, C)** Principal Component Analysis (PCA) of RA and control samples. **(D)** Volcano plot illustrating the distribution of DEGs. **(E)** Heatmap visualizing the expression patterns of DEGs.

The research workflow is illustrated in **Figure 1**. Data standardization is shown in a box plot, where various colors represent distinct data sets, with rows corresponding to samples and columns indicating gene expression values (**Figure 2A**). **Figure 2B** demonstrates the PCA results from multiple data sets before addressing batch effects, using different colors to differentiate the data sets. **Figure 2C** shows the PCA results after batch effect removal, indicating that the integration of the three data sets allows for collective analysis. Applying the standards of |logFC| > 1 and an adjusted *P*-value < 0.05, 543 genes were found to be DEGs, of which 220 were down-regulated and 323 were up-regulated. **Figure 2D** shows the volcano plots of DEGs, and **Figure 2E** shows a heat map of the top 50 genes.

### Data collection and module analysis

**Figure 3:**
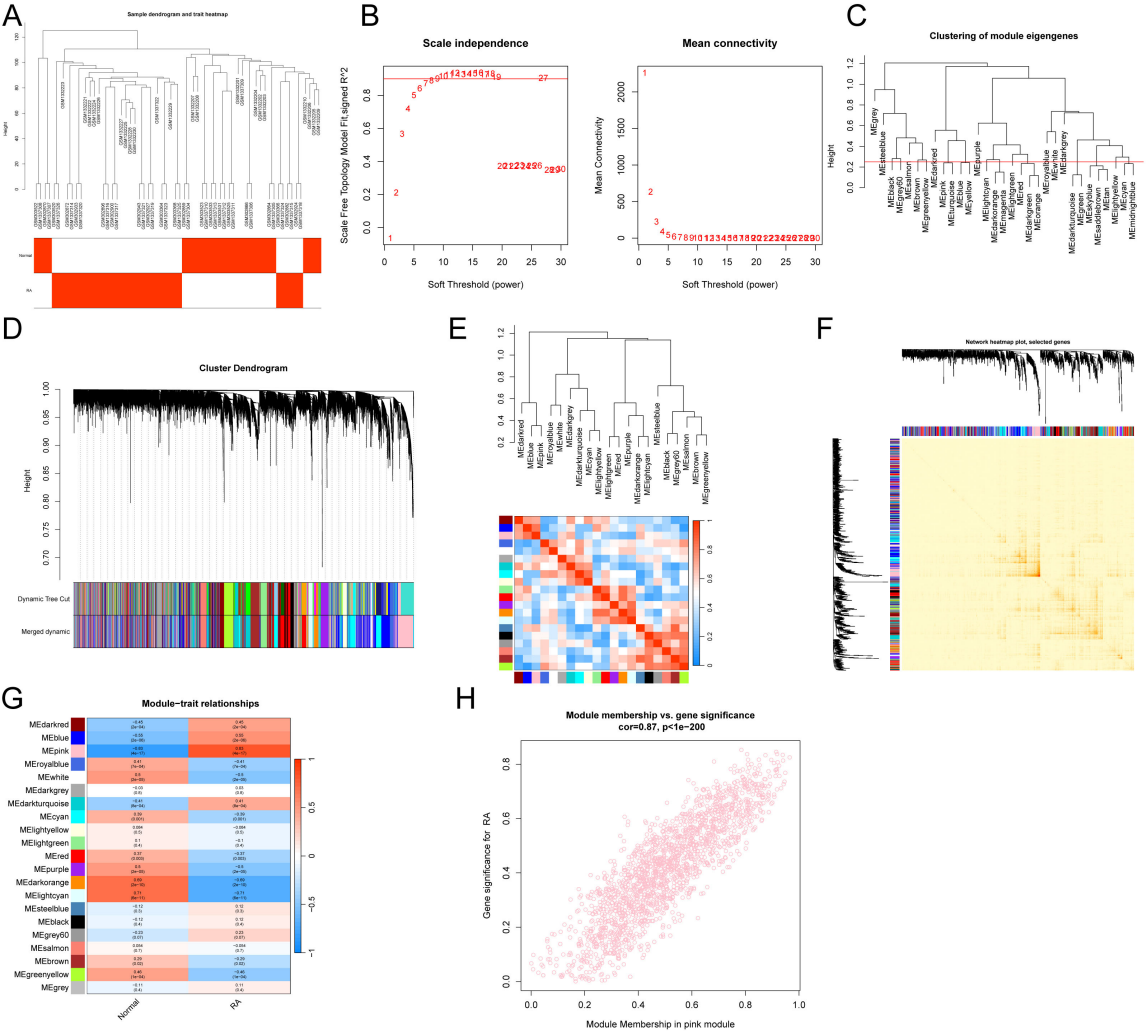
Establishing the WGCNA network. **(A)** Sample clustering dendrogram: individual samples are represented by the leaves of the trees. **(B)** Selecting a soft threshold, β = 8, is necessary to get a scale-free topological fit index (R^2^). **(C)** Clustered dendrograms with a 0.25-height cut to help find and combine related modules. **(D)** Original and merged modules are shown below the tree of clusters. **(E)** A collinear heatmap displays the genes that comprise the module features; blue indicates inverse associations, and red denotes strong correlation. **(F)** Module feature gene clustering dendrogram. **(G)** A heatmap showing the relationships between module traits; positive correlations are shown in red, while negative correlations are indicated in blue. **(H)** Scatter plot showing the relationship between the controls’ gene significance (GS) and module membership (MM).

Datasets GSE12021, GSE55457, and GSE55235 were obtained from the GEO database, consisting of 29 normal and 35 RA samples. These samples were clustered using WGCNA to remove obvious outliers, with a defined threshold (**Figure 3A**). **Figure 3B** shows a soft-threshold power of 8, with an R^2^ > 0.9 and strong average connectedness. A clustering height limit of 0.25 was set to merge strongly related modules, resulting in 21 modules for further analysis (**Figure 3C**). These modules were displayed on the clustering tree (**Figure 3D**). Module correlation analysis showed no significant associations among them (**Figure 3E**). The reliability of the module delineation was validated by transcription correlation analysis within the modules, which also revealed no significant connections (**Figure 3F**). To explore the relationship between modules and clinical features, correlations between module eigengene (ME) values and clinical features were analyzed. The pink module showed a positive correlation with normal samples (*r* = 0.83, *P* = 4e−17) and a negative correlation with RA samples (*r* = -0.83, *P* = 4e−17) (**Figure 3G**). This module, strongly associated with RA, was identified as clinically relevant, as shown in the MM vs. GS scatter plot (**Figure 3H**). Further analysis was then performed on all genes within the pink module.

### Functional analysis of critical module genes and DEGs

**Figure 4:**
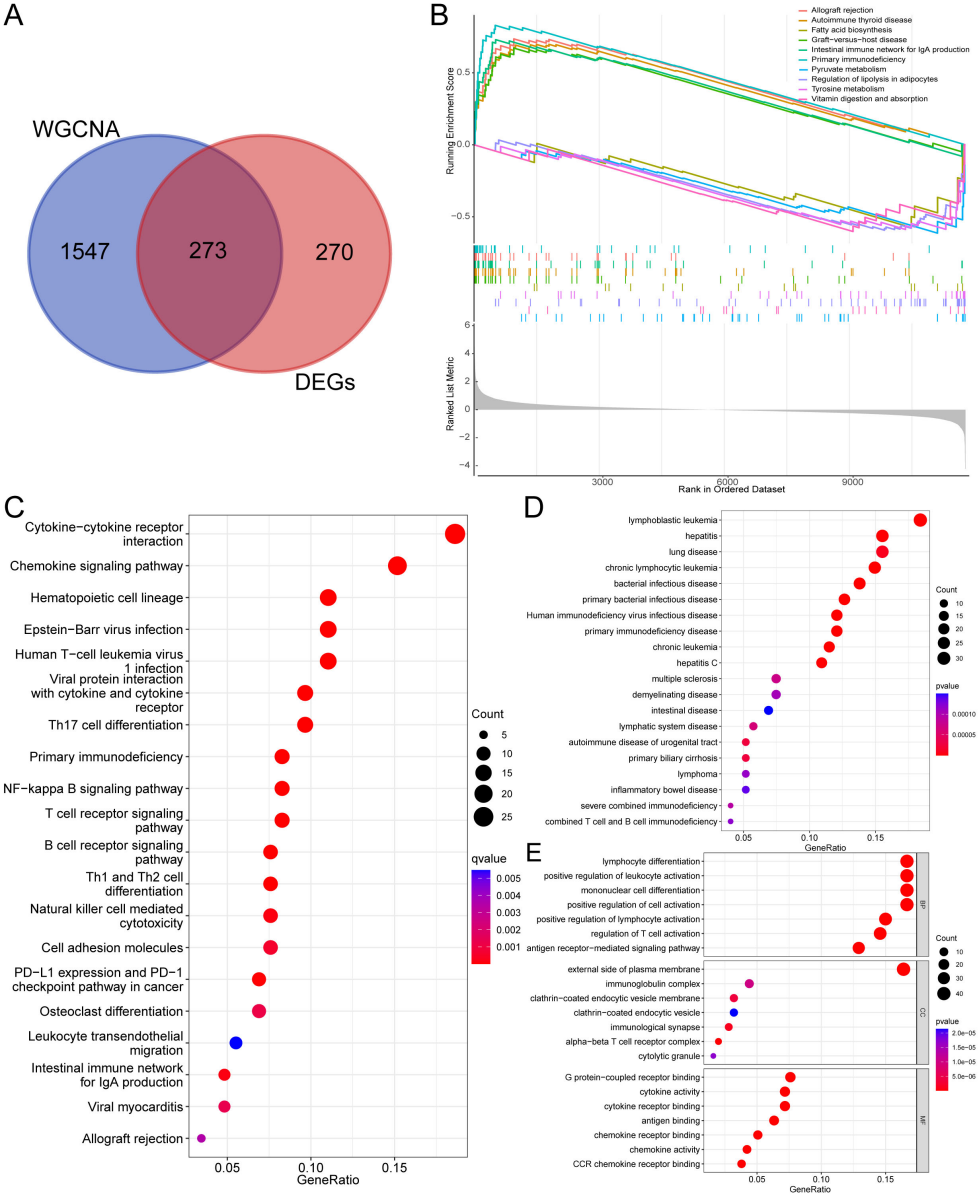
Functional evaluation of important module genes that are combined with DEGs. **(A)** Venn diagram showing the DEGs and important module genes. **(B)** GESA analysis. **(C)** KEGG pathway analysis. **(D)** DO analysis. **(E)** GO analysis.

A Venn diagram was used to overlap critical module genes with DEGs, resulting in the identification of 273 overlapping genes (**Figure 4A**). Functional analysis elucidated the biological functions of these DEGs within the modules. GSEA results indicated that these DEGs are associated with allograft rejection, autoimmune thyroid disease, pyruvate metabolism, and tyrosine metabolism (**Figure 4B**). KEGG analysis linked these DEGs to cytokine-cytokine receptor interactions, Th17 cell differentiation, chemokine signaling, and T cell receptor signaling pathways (**Figure 4C**). DO analysis demonstrated involvement of these DEGs in lymphoblastic leukemia, hepatitis, lung diseases, and chronic lymphocytic leukemia (**Figure 4D**). GO enrichment analysis revealed that the module DEGs enhance lymphocyte activation, mononuclear cell differentiation, leukocyte activation, T cell activation, and activities of chemokines, cytokines, and G protein-coupled receptors (**Figure 4E**).

### Selection of feature genes

**Figure 5:**
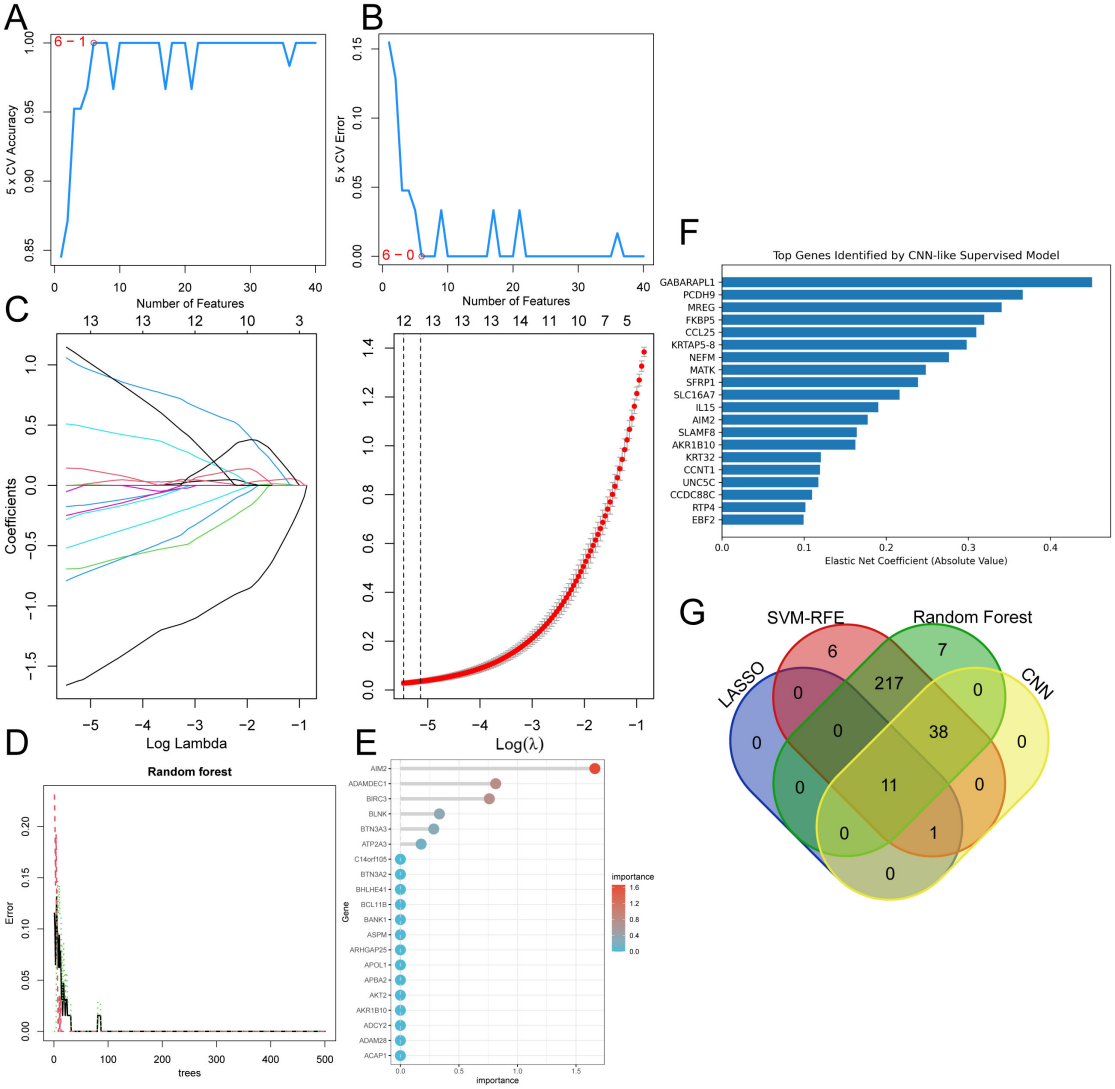
Selection of feature genes. **(A, B)** Validation of gene expression associated with biomarker signatures using the SVM-RFE method. **(C)** Optimization of feature selection through the LASSO model. **(D)** Examination of error rates in Random Forest with varying numbers of classification trees. **(E)** Identification of the twenty most important genes. **(F)** CNN analysis results, displaying the top 20 genes. **(G)** Feature genes derived from four machine learning techniques were screened using a Venn diagram.

To identify feature genes, we utilized key module genes from WGCNA analysis and 273 core genes derived from the intersection of DEGs as inputs for four advanced machine learning algorithms: LASSO regression, RandomForest, SVM-RFE, and CNN. These algorithms classified genes based on their ability to distinguish RA samples from healthy controls. The results of the SVM-RFE analysis are shown in **Figures 5A, B**, with the associated gene list provided in **Supplementary Table 2**. LASSO regression identified twelve genes based on statistically significant univariate factors, as shown in **Figure 5C** and **Supplementary Table 3**. RandomForest was used to assess the error rate, the number of classification trees, and the relevance of the 273 genes, incorporating feature selection. The results of this analysis are shown in **Figures 5D, E** with the relevant gene list available in **Supplementary Table 4**. The Performance Metrics of Machine Learning Models for RA Biomarker Prediction are included in **Supplementary Table 5**. To complement these methods, CNN was applied for further analysis. CNN captured complex gene expression patterns and improved classification accuracy. The CNN results, including the most significant feature genes, are presented in **Figure 5F** and detailed in **Supplementary Table 6**. Finally, a Venn diagram (**Figure 5G**) was used to identify eleven overlapping genes shared by all four methods, highlighting the most consistently identified feature genes.

### Modeling and testing of an RA diagnostic nomogram

**Figure 6:**
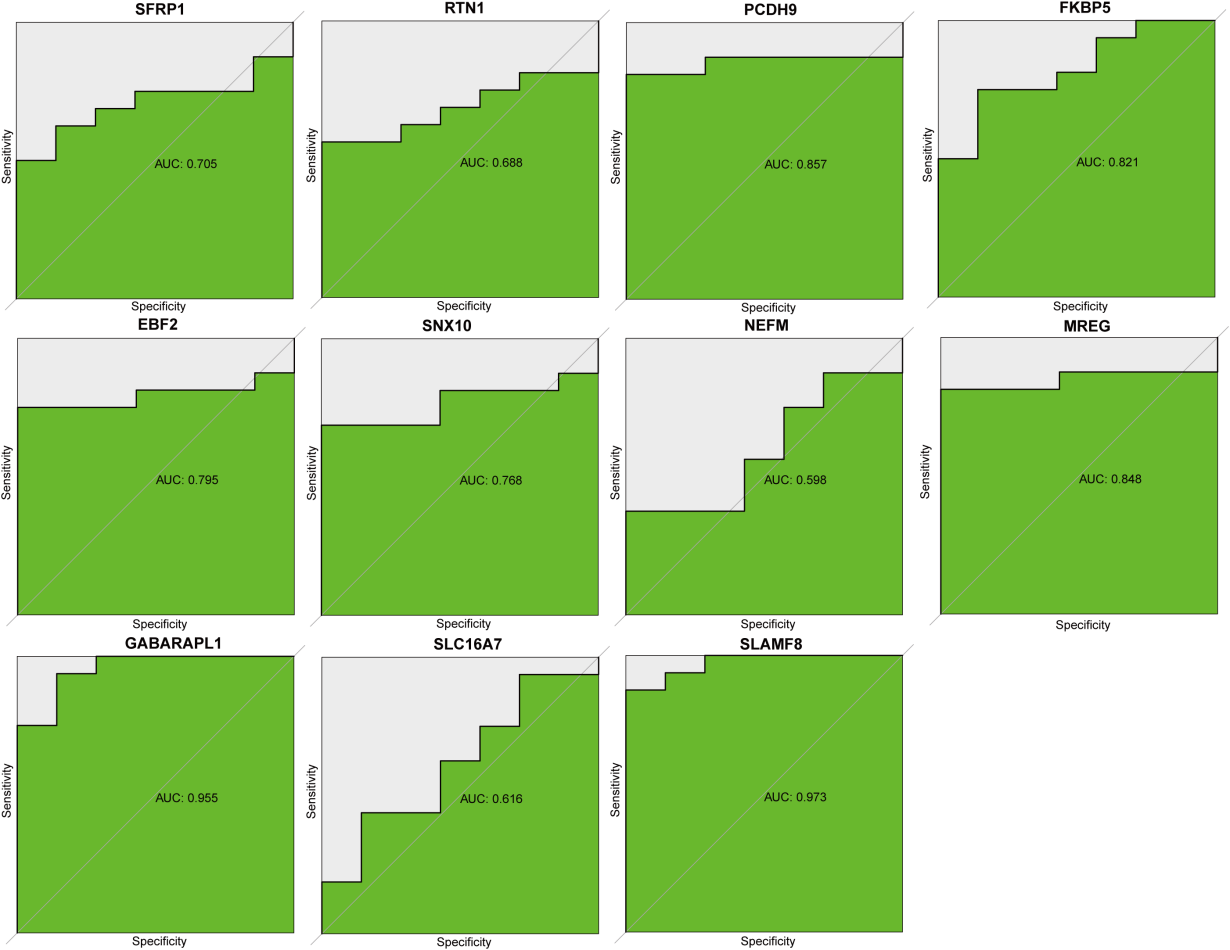
ROC curve analysis for the hub genes.

**Figure 7:**
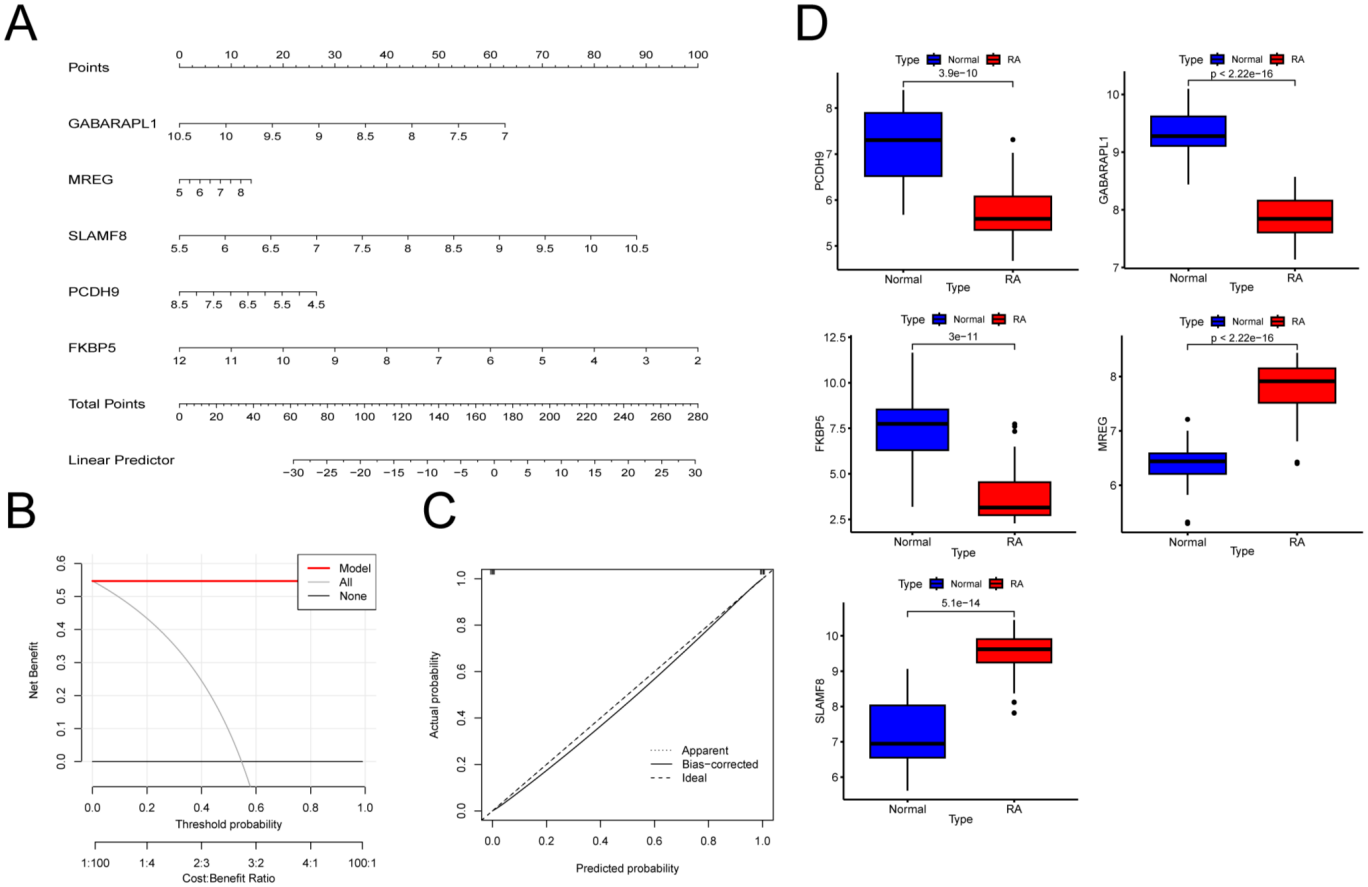
Development and validation of the RA diagnostic columnar line graph model. **(A)** Columnar line graphs used to predict RA incidence. **(B)** ROC curve analysis to evaluate the clinical utility of the columnar line graph model. **(C)** DCA curve analysis to assess the clinical utility of the columnar line graph model. **(D)** Box plot illustrating the expression levels of GABARAPL1, FKBP5, MREG, PCDH9, and SLAMF8.

To evaluate the diagnostic efficacy of EBF2, FKBP5, GABARAPL1, MREG, NEFM, PCDH9, RTN1, SFRP1, SLAMF8, SLC16A7, and SNX10, ROC analysis was conducted. The AUC values obtained were: EBF2 (0.795), FKBP5 (0.821), GABARAPL1 (0.955), MREG (0.848), NEFM (0.598), PCDH9 (0.857), RTN1 (0.688), SFRP1 (0.705), SLAMF8 (0.973), SLC16A7 (0.616), and SNX10 (0.768) (**Figure 6**). The five genes with the highest AUC values—FKBP5, GABARAPL1, MREG, PCDH9, and SLAMF8—were used to develop nomogram models for RA diagnostics using the “rms” package (**Figure 7A**). Their predictive accuracy was evaluated using calibration curves. Calibration curves showed minimal differences between actual and predicted RA risks, indicating high accuracy of the nomogram models (**Figure 7B**). The model’s accuracy was further verified through additional ROC curve analysis (**Figure 7C**). Validation with datasets GSE12021, GSE55235, and GSE55457 corroborated these findings (**Figure 7D**). These findings suggest that the five key genes are involved in the pathogenesis of RA.

### Trait gene interaction analysis

**Figure 8:**
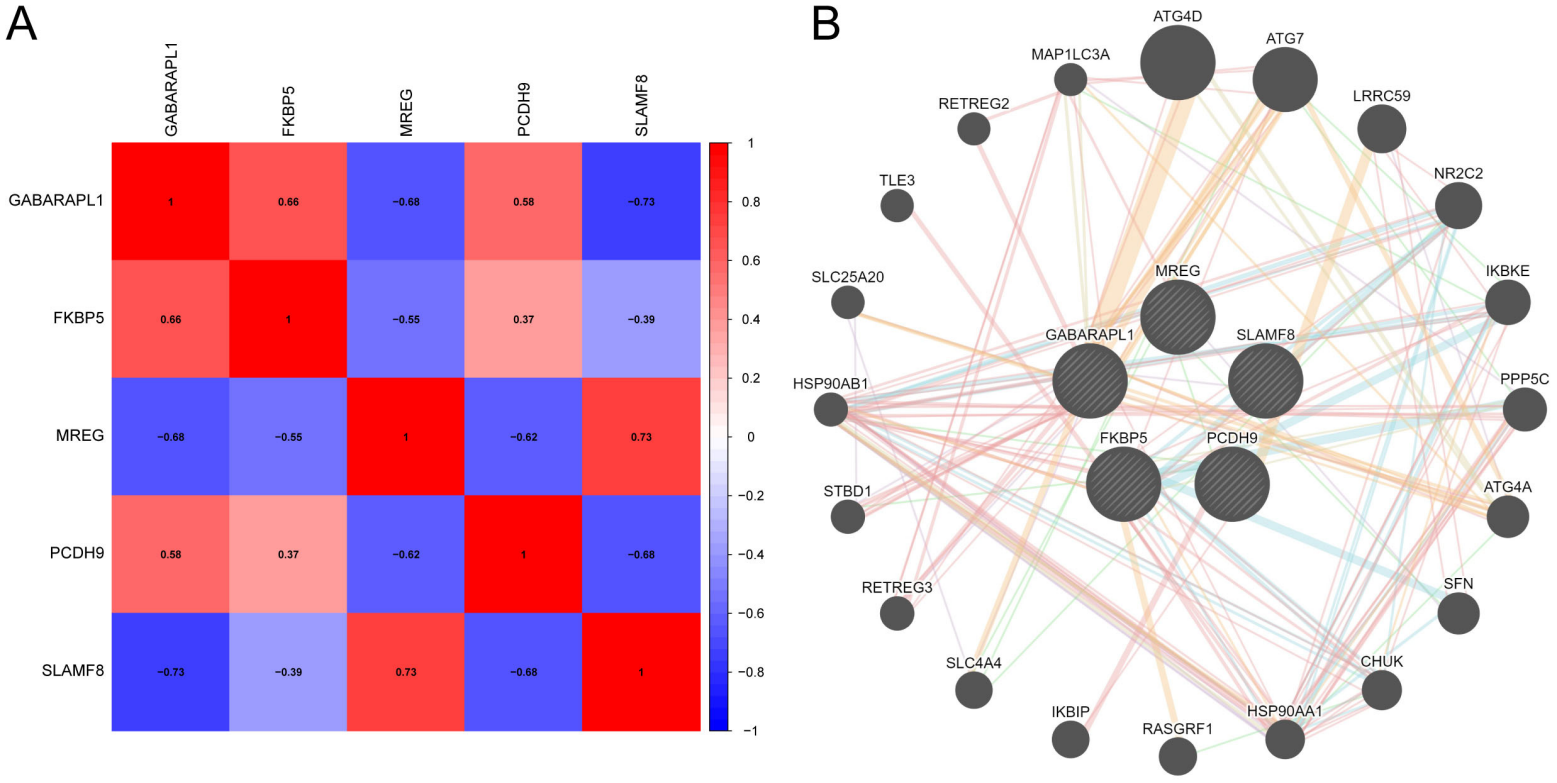
Analysis of interactions among feature genes. **(A)** Correlation analysis between hub genes. **(B)** Construction of a co-expression network for hub genes.

Gene correlations were examined, revealing both positive and negative correlations among the expressions of FKBP5, GABARAPL1, MREG, PCDH9, and SLAMF8, as depicted in **Figure 8A**. This indicates significant functional relationships among these five genes. Subsequently, using the online platform GeneMANIA (http://genemania.org/), an intuitive network diagram was created to illustrate the interactions and relationships among these genes, highlighting their closely linked functions (**Figure 8B**).

### ssGSEA analysis and correlation analysis

**Figure 9:**
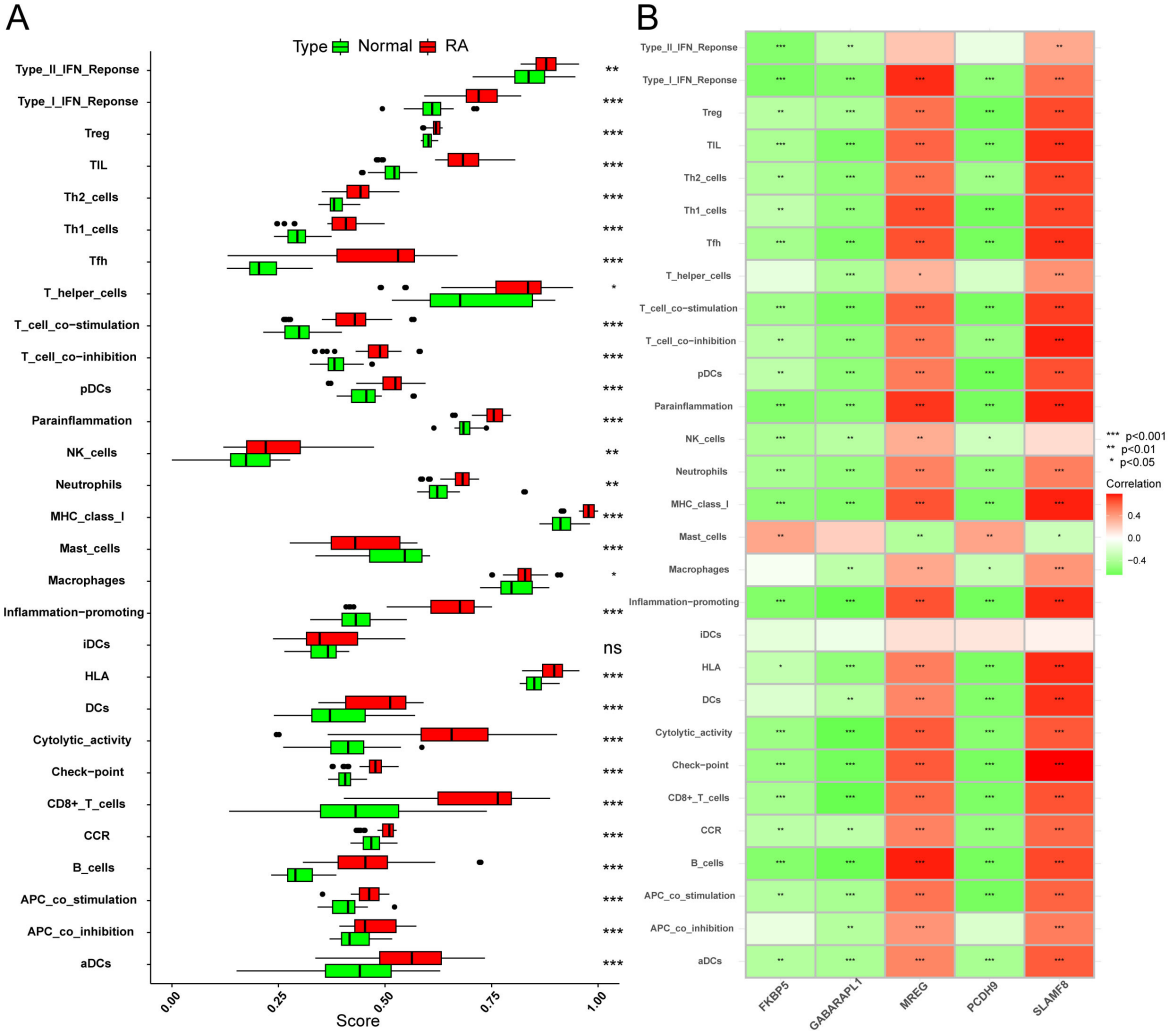
Investigation of the relationship between immunological response and RA. **(A)** Comparison of the RA group’s and the healthy controls’ ssGSEA scores for immune cells and pathways. **(B)** Analysis of the correlation between immunological responses and distinctive genes linked to immunity. The following is an indication of statistical significance: *P < 0.05, **P < 0.01, *P < 0.001, ns (not significant).

Using ssGSEA, the relationship between immune infiltration in RA patients and healthy controls was investigated further. When statistically non-significant data were removed from the analysis, it was shown that although immune cell infiltration in mast cells was lower in RA patients than in controls, other immune-related pathway activities were higher in the overall RA group (**Figure 9A**). Subsequently, the “corrplot” package was utilized to analyze the correlation between signature genes and immune infiltration. FKBP5, GABARAPL1, and PCDH9 exhibited strong negative correlations with several immune functions, including CD8+ T cells, checkpoints, cytolytic activity, inflammation promotion, MHC class I, neutrophils, T-cell co-stimulation, Tfh, Th1/2 cells, TIL, Treg, and Type I IFN response. Conversely, MREG and SLAMF8 demonstrated strong positive correlations with functions such as Type I IFN response, Tfh, TIL, Th1/2 cells, T-cell co-inhibition and co-stimulation, checkpoints, and cytolytic activity (**Figure 9B**). These results imply that the hallmark genes may modify immunological mechanisms along the course of RA.

### Validation of the hub genes expression

**Figure 10:**
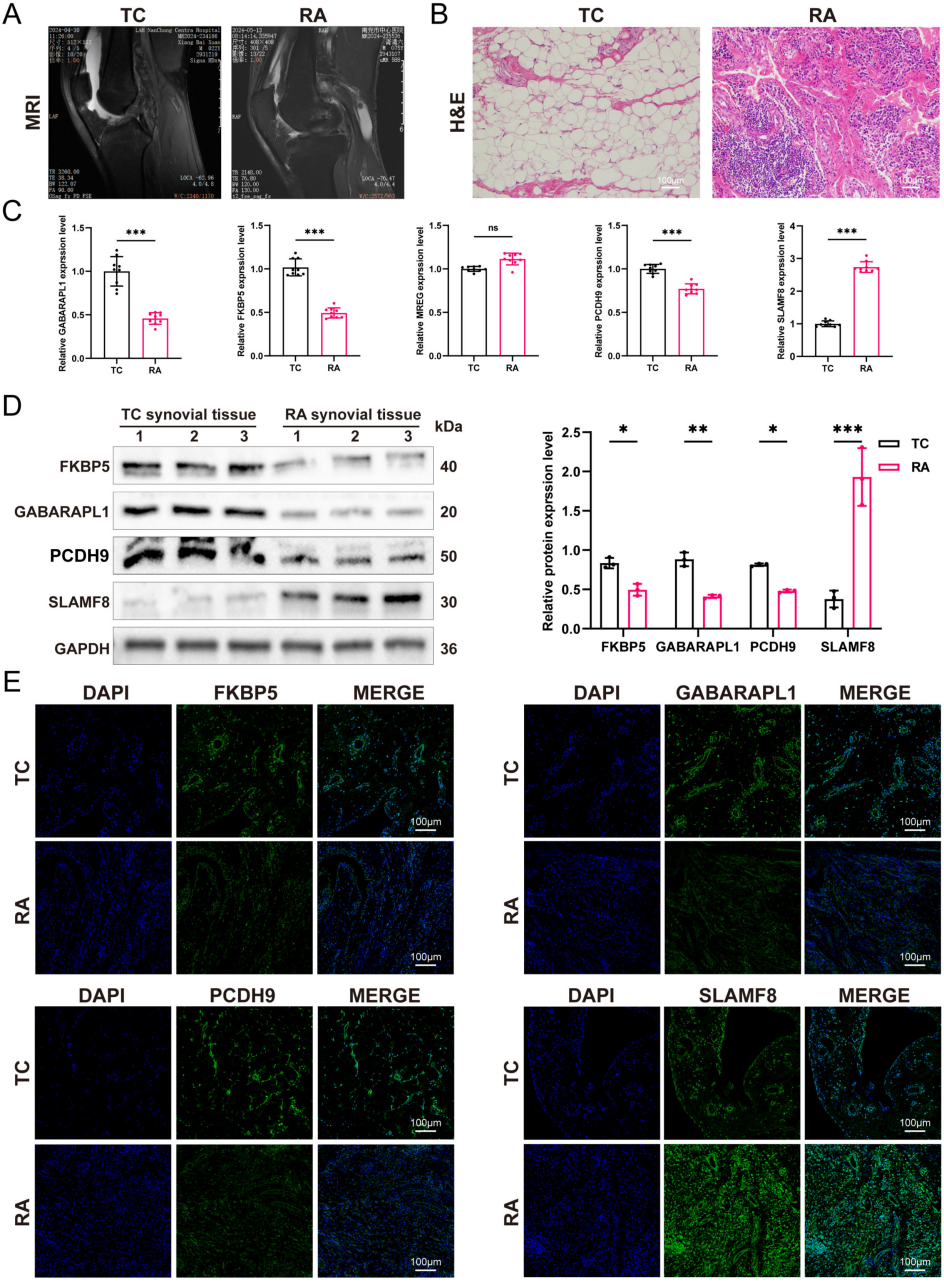
Analysis of hub gene expression in synovial tissues from RA patients. **(A)** Representative MRI scans illustrating synovial thickening. **(B)** H&E staining of synovial tissue in trauma control (TC) and RA samples (n = 3). **(C)** Comparative analysis of mRNA expression levels for GABARAPL1, FKBP5, MREG, PCDH9, and SLAMF8 in TC and RA samples (n = 9). **(D)** Quantitative assessment of protein concentrations for GABARAPL1, FKBP5, PCDH9, and SLAMF8 in TC and RA samples (n = 3, the samples derive from the same experiment and that gels/blots were processed in parallel. original blots/gels are presented in **Supplementary Figure 1**). **(E)** IF staining results showed protein expression of GABARAPL1, FKBP5, PCDH9, and SLAMF8 in TC and RA samples, visualized with DAPI (blue) and specific antibodies (green) (n = 3). The means ± SD are used to represent the data, while ns (not significant), *P < 0.05, **P < 0.01, and *P < 0.001 indicate significance levels.

**Figure 11:**
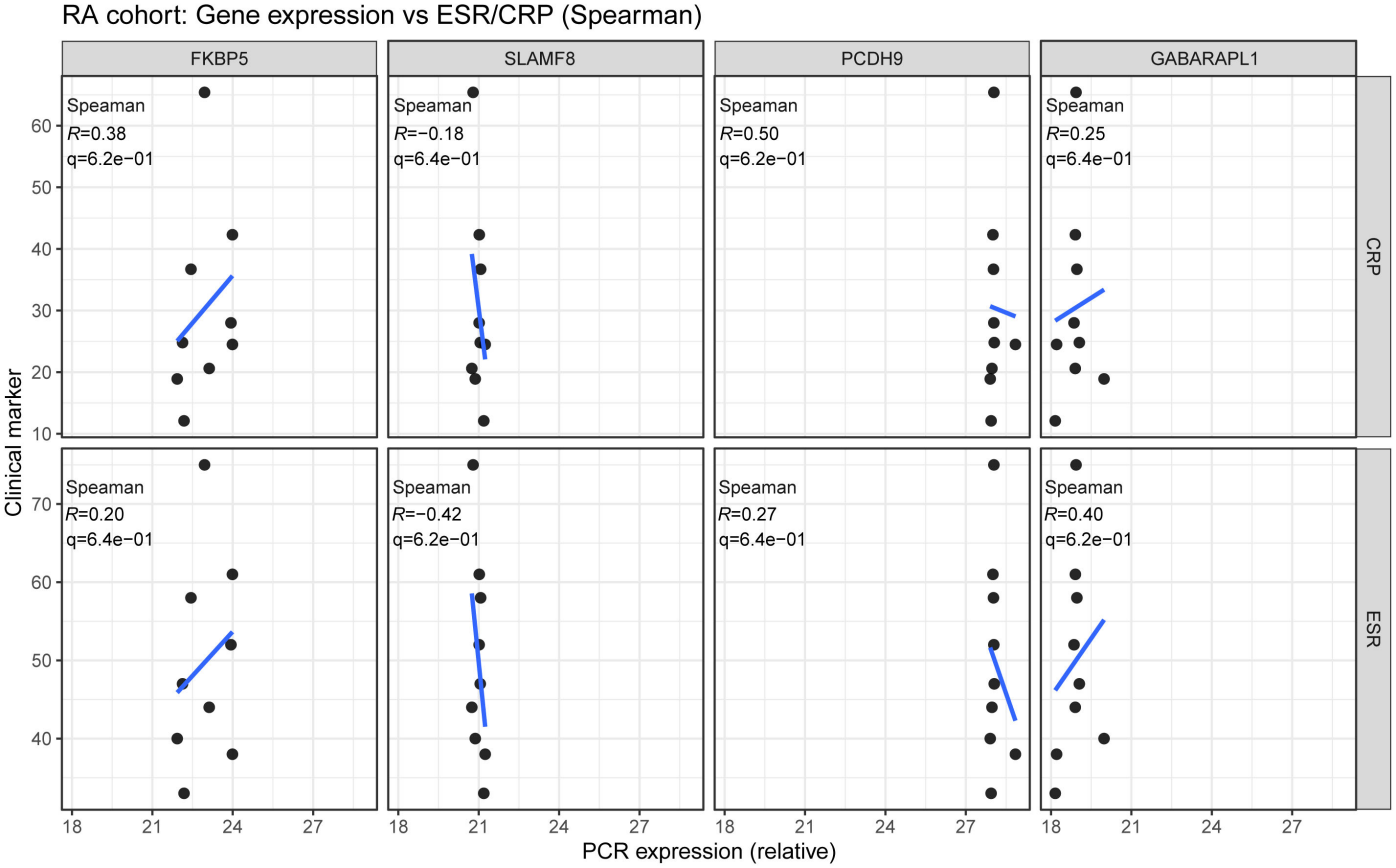
Correlation between candidate gene expression and inflammatory indices in the RA cohort. Scatter plots show the associations between PCR-measured expression levels of the candidate genes (GABARAPL1, FKBP5, PCDH9, and SLAMF8) and serum inflammatory markers (ESR and CRP) within RA patients only (n = 9). Spearman’s rank correlation was used to estimate the correlation coefficient (*R*), with *p* values adjusted for multiple testing using the Benjamini–Hochberg false discovery rate (FDR).

To identify potential biomarkers for RA, we selected five genes—FKBP5, GABARAPL1, MREG, PCDH9, and SLAMF8—based on their strong diagnostic potential and involvement in immune regulation, as indicated by previous bioinformatics analyses, suggesting their role in the disease’s development. Synovial tissues from nine patients diagnosed with RA and nine trauma control (TC) subjects were collected. Magnetic resonance imaging (MRI) analysis revealed synovial thickening in RA patients compared to the TC group (**Figure 10A**). H&E staining of RA synovial tissues indicated enhanced cellular density and heterogeneity in the intercellular matrix. Notably, significant increases in both the number and staining intensity of cell nuclei were observed, indicating elevated cell proliferation and substantial inflammatory cell infiltration (**Figure 10B**). qRT-PCR analyses demonstrated decreased expression of FKBH5, GABARAPL1, and PCDH9, and increased expression of SLAMF8 in the RA synovium; the rise in MREG expression was not statistically significant (**Figure 10C**). Western blot and IF analyses confirmed the downregulation of FKBH5, GABARAPL1, and PCDH9, along with the upregulation of SLAMF8, in the RA group compared to the TC group (**Figures 10D, E**). Spearman’s rank correlation analysis showed that FKBP5, GABARAPL1, and PCDH9 expression levels were negatively correlated with inflammatory activity, whereas SLAMF8 exhibited a positive correlation (**Figure 11**). All of these results show that the expression levels of FKBH5, GABARAPL1, PCDH9, and SLAMF8 in RA synovium vary significantly from those in TC synovium.

### Identification of candidate drugs

Understanding the structural factors influencing receptor sensitivity requires examining protein–drug interactions (31, 32). In this study, we used transcriptomic data from the DSigDB database and the Enrichr tool to identify 244 candidate pharmacological compounds associated with hub genes that may be relevant to RA. While these compounds have shown promise in in silico analyses as candidates for modulating hub gene activity, further validation and experimental studies are necessary to assess their potential role in RA treatment. The top ten compounds identified from the DSigDB database, ranked by *P*-values, are listed in **Supplementary Table 7**. It is important to note that many of these compounds, such as daunorubicin, cupric oxide, and methaneseleninic acid, are cytotoxic or have been studied in contexts other than RA. Therefore, the current evidence supporting their relevance to RA is computational and should be considered hypothesis-generating.

## Discussion

Rheumatoid arthritis (RA) severely impairs the quality of life for sufferers and causes irreparable damage to the synovial lining of joints (33). The incidence of RA has grown during the last several decades despite advancements in therapy techniques (34). Early diagnosis and treatment of RA are crucial for preventing joint damage and improving quality of life. However, the lack of effective biomarkers makes diagnosing early-stage RA challenging. Identifying novel, reliable biomarkers is essential for RA therapy and early diagnosis. The purpose of this research was to find new biomarkers for RA diagnosis and treatment and to look at how they relate to immune infiltration. Several bioinformatics techniques and *in vivo* tests were used to identify and verify four diagnostic potential diagnostic biomarkers for RA. These four diagnostic potential diagnostic biomarkers showed notable variations in expression within the immune microenvironment.

In this study, we identified 543 DEGs by comparing gene expression in samples from the healthy controls and RA groups. We then performed WGCNA analysis, which revealed 21 co-expression modules. Genes from modules highly correlated with RA phenotypes were selected and intersected with the DEGs, resulting in 273 key genes. A subsequent GO enrichment analysis indicated that these key genes were predominantly associated with cytokine receptor function and the activation of inflammatory cells. Additionally, KEGG enrichment analysis demonstrated their involvement in T-cell receptor signaling, chemokine signaling, and cytokine-cytokine receptor interactions. The enrichment analysis further revealed that RA synovium exhibits strong immune activation and signal transduction effects, which play major roles in RA-related synovial inflammation, arthritis, and joint pain. Arthritis and joint pain are well-established as the primary clinical manifestations of RA (35).

Machine learning provides an objective approach for predicting patient status, simultaneously revealing previously unknown interactions and identifying novel biological signatures (36, 37). Using four machine learning algorithms and ROC curves, we identified GABARAPL1, FKBP5, MREG, PCDH9, and SLAMF8 as diagnostic signature biomarkers. As demonstrated by the diagnostic nomogram, these five RA biomarkers possess significant diagnostic value. Furthermore, clinical tissue validation data showed notable differences in the expression of four diagnostic biomarkers (GABARAPL1, FKBP5, PCDH9, and SLAMF8) between TC and RA tissues. Immune infiltration analysis also revealed strong correlations between these biomarkers and various immune cell functions, as well as immune-related pathways. Finally, we identified potential drugs targeting these four DEGs, including (+)-chelidonine, daunorubicin, and bisacodyl. Investigating the interactions between these key genes and their respective drugs could significantly contribute to advancing drug development for RA. Therefore, based on our findings, we propose that GABARAPL1, FKBP5, PCDH9, and SLAMF8 may serve as biomarkers for the early diagnosis of RA.

GABARAPL1 is a member of the GABARAP family and is also known as autophagy-related 8 (ATG8) or glandular epithelial cell protein 1 (GEC1) (38). In addition to being engaged in the movement of proteins and vesicles, the GABARAP group members (GABARAP, GABARAPL1, and GABARAPL2) and their homologs (LC3 and Atg8) are also involved in many other processes, including autophagy, apoptosis, cell development, and cancer (38). GEC1 regulates mitochondrial homeostasis, which is necessary for autophagosome maturation, as An et al. (39) showed. Further evidence that GEC1 maintains appropriate autophagic flow and is essential for controlling cell bioenergetics and metabolism was provided by Boyer-Guittaut et al. (40). GABARAPL1 may affect cellular energy and cytoskeletal reorganizations required for immune cell migration under its function in autophagy (41). Research suggests that GABARAPL1 may indirectly influence the threshold for immune cell death by regulating the autophagic response. For instance, increased autophagic activity mediated by GABARAPL1 might lessen the tendency for cell apoptosis and increase cell survival under stress (42, 43). According to our research, the RA synovium has downregulated GABARAPL1 expression. Furthermore, GABARAPL1’s ROC curve demonstrated that it has a strong diagnostic value for RA (AUC = 0.955). We believe that GABARAPL1 is a useful biomarker for the diagnosis of RA.

FKBP5, also known as FKBP51, is a member of the FK506 binding protein (FKBP) family and is involved in many biological functions, including transport, immunological control, and protein folding (44, 45). FKBP51 functions as a chaperone protein, affecting the production of proinflammatory cytokines by modulating the transcription factor nuclear factor kappa B (NF-κB) (46). Fascinatingly, Nakamura et al. (47) recently reported that RA patients’ bone marrow (BM) mononuclear cells had elevated expression levels of many genes, including FKBP5. Nonetheless, we discovered that the RA synovium had downregulated FKBP5 expression. Consequently, FKBP5 could be important for the development of RA illness.

PCDH9 belongs to the biggest subfamily in the cadherin group, the protocadherin family (48). A member of the δ1-subfamily that also contains PCDH1, PCDH7, PCDH9, and PCDH11, PCDH9 is involved in the formation and breakdown of cell adhesions (49). Protocadherins influence cell migration, survival, and growth inhibition, all of which are important aspects of carcinogenesis (50). Due to their excessive activation and tumor-like features, FLSs play a critical role in the onset and evolution of RA by producing matrix metalloproteinase (MMP), pro-inflammatory cytokine release, invasion, and aberrant proliferation (51–54). Thus, we speculate that PCDH9 plays a role in developing features resembling tumors in synoviocytes. Furthermore, we discovered that PCDH9 had a good diagnostic value for RA (AUC = 0.857) and was only weakly expressed in the synovium of RA patients. In our opinion, PCDH9 is a new and useful biomarker for diagnosing RA.

The signaling lymphocytic activation molecule family (SLAMF) is a family of surface receptors that regulate the activity of immune cells and is extensively expressed in hematopoietic cells, including T cells, NK cells, and macrophages (55). It has been shown that SLAMF8, a member of the SLAMF family, lowers ROS generation and inhibits macrophage activity (56). Additionally, it has been shown to be a noteworthy indicator of T-cell infiltration into malignancies (57). The TLR4/NF-κB signaling pathway was blocked by targeted suppression of SLAMF8, which decreased the severity of RA (58). Furthermore, we discovered that SLAMF8 had a strong diagnostic value for RA (AUC = 0.973) and was substantially expressed in the synovium of RA patients. Therefore, we think SLAMF8 is a useful biomarker for RA diagnosis.

## Limitations

This research has certain limitations, even though our nomogram model, which is based on five distinctive genes, shows strong diagnostic predictive capacity for RA patients. First of all, certain demographic and clinical characteristics (e.g., sex, age, comorbidities) were not systematically evaluated in this database-derived study, highlighting the need for further clinical investigations to validate these findings. Secondly, the biological functions of the genes that have been discovered and their associations with RA remain incompletely understood. Lastly, biased validation data arise from the causes of clinical sample limits. To further confirm the practical applicability value of our nomogram model in identifying RA patients, we want to increase clinical patient recruitment.

## Conclusion

In conclusion, we found that FKBP5, GABARAPL1, PCDH9, and SLAMF8 are diagnostic biomarkers for RA linked to immune infiltration via our investigation using a machine learning method. These results provide a fresh viewpoint on the pathophysiology of RA.

## Data Availability

The NCBI GEO repository has the datasets that this study is based on: GSE12021, GSE55235, GSE55457, GSE77298, and https://www.ncbi.nlm.nih.gov/geo/. The corresponding author may provide the experimental data utilized in this work upon reasonable request.
