# Supplementary figures and images for: Unveiling potential diagnostic biomarkers for rheumatoid arthritis through integrated gene expression analysis
Source: Front Immunol. 2026 Feb 24;17:1645257. doi: 10.3389/fimmu.2026.1645257 (PMC12971669; doi:10.3389/fimmu.2026.1645257)

The original blots in Figure 10 are presented below.

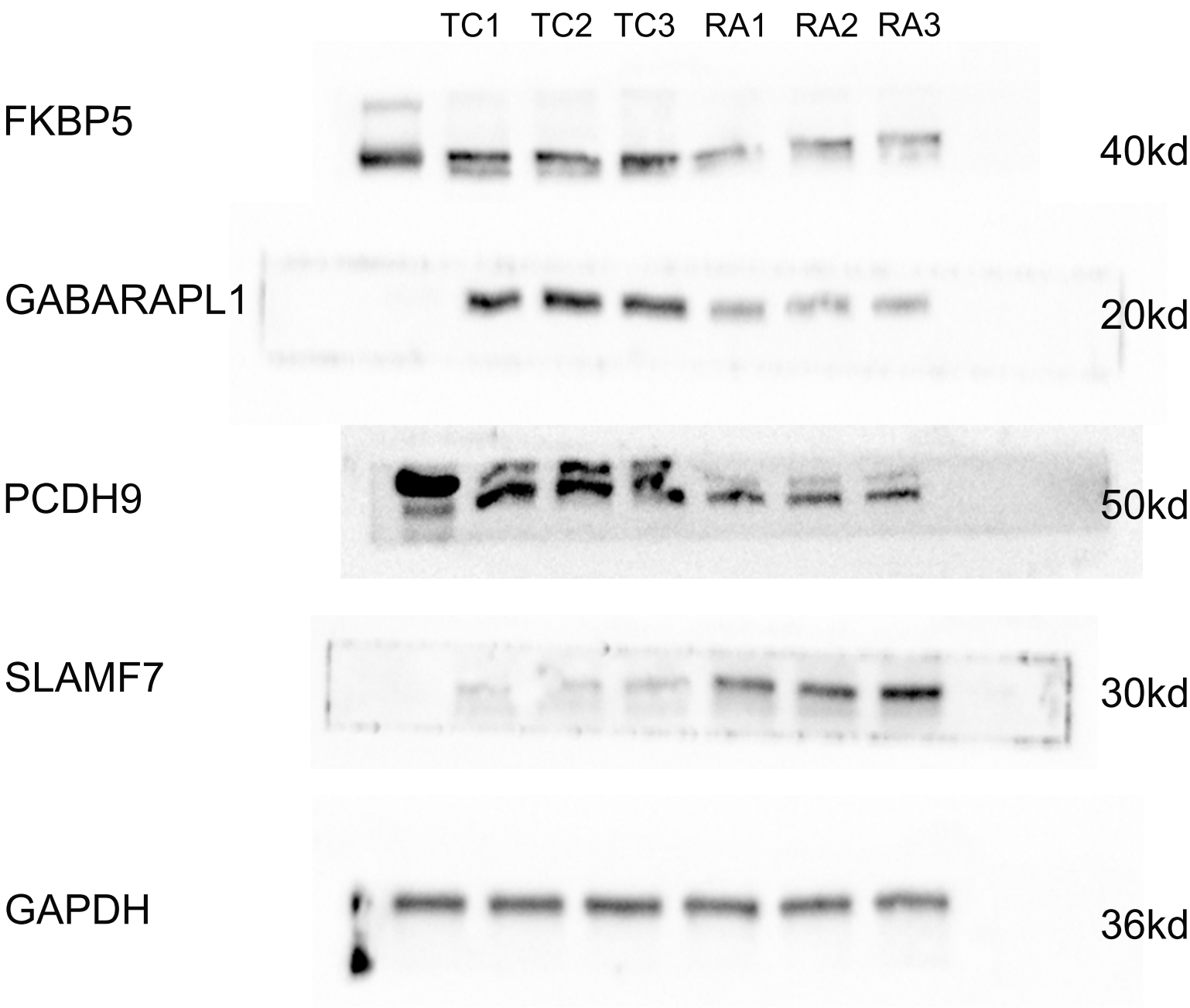

Supplement: Supplementary file 2 [file DataSheet1.pdf]
